# On the Genetic Interpretation of Disease Data

**DOI:** 10.1371/journal.pone.0008940

**Published:** 2010-01-28

**Authors:** Stephen C. Bishop, John A. Woolliams

**Affiliations:** The Roslin Institute and Royal (Dick) School of Veterinary Studies, University of Edinburgh, Roslin, Midlothian, United Kingdom; Health Canada, Canada

## Abstract

**Background:**

The understanding of host genetic variation in disease resistance increasingly requires the use of field data to obtain sufficient numbers of phenotypes. We introduce concepts necessary for a genetic interpretation of field disease data, for diseases caused by microparasites such as bacteria or viruses. Our focus is on variance component estimation and we introduce epidemiological concepts to quantitative genetics.

**Methodology/Principal Findings:**

We have derived simple deterministic formulae to predict the impacts of incomplete exposure to infection, or imperfect diagnostic test sensitivity and specificity on heritabilities for disease resistance. We show that these factors all reduce the estimable heritabilities. The impacts of incomplete exposure depend on disease prevalence but are relatively linear with the exposure probability. For prevalences less than 0.5, imperfect diagnostic test sensitivity results in a small underestimation of heritability, whereas imperfect specificity leads to a much greater underestimation, with the impact increasing as prevalence declines. These impacts are reversed for prevalences greater than 0.5. Incomplete data recording in which infected or diseased individuals are not observed, e.g. data recording for too short a period, has impacts analogous to imperfect sensitivity.

**Conclusions/Significance:**

These results help to explain the often low disease resistance heritabilities observed under field conditions. They also demonstrate that incomplete exposure to infection, or suboptimal diagnoses, are not fatal flaws for demonstrating host genetic differences in resistance, they merely reduce the power of datasets. Lastly, they provide a tool for inferring the true extent of genetic variation in disease resistance given knowledge of the disease biology.

## Introduction

Genetic variation in host resistance to infectious disease is ubiquitous [1], [2], [3]. The increasing realization of this phenomenon has led to disease biology becoming a major focus of ecology and population or quantitative genetic research for human and animal geneticists alike. Further, the ready availability of dense single nucleotide polymorphism arrays (i.e. SNP chips) has given rise to hitherto unforeseen opportunities to dissect this between-host variation and identify possible genes contributing to this variation using genome wide association studies [4]. This, coupled with more traditional quantitative genetic variance-partitioning approaches [5], enables detailed descriptions of genetic aspects of disease resistance and the identification of individuals with extreme (high or low) risk of infection or disease [6]. Such techniques can be applied equally to human, natural animal populations or farmed livestock.

To have the requisite power to meaningfully quantify genetic variation or perform a genome scan using a dense SNP chip it is necessary to have datasets comprising observations on several thousands of individuals [e.g. 7]. For studies of infectious diseases this usually necessitates utilizing field data because challenge experiments of a sufficient scale will not be possible, possibly excepting studies with aquacultural species [e.g. 8]. For example, in the livestock context, data may be captured from a population undergoing an epidemic such as bovine tuberculosis [9], or from an endemic disease such as mastitis [see 10], where the herd-level prevalence is largely predictable. However, such field data is very ‘noisy’: diagnosis of infection or disease may be imprecise; it can be difficult to determine when infection of an individual occurred; and it is often unclear whether or not apparently healthy individuals have been exposed to the infection. These factors will add environmental noise to the epidemiological data.

Issues such as exposure and diagnostic test sensitivity or specificity are fundamental concepts to epidemiologists when studying the spread of disease in a population [11], yet their intrinsic importance is currently ignored in quantitative genetic theory [5]. Quantifying and accounting for the impact of environmental factors is an integral part of identifying and measuring true host genetic variation in resistance to the disease under study. Consequently, there is an unrecognised risk of biases in genetic parameter estimates and lost opportunities for identifying individuals with extreme genetic risk. This paper proposes advances in quantitative genetic theory using concepts borrowed from epidemiology and provides predictive equations for the impact of epidemiological factors on heritability estimation. The theory is developed specifically for microparasitic infections, such as those caused by viruses or bacteria.

## Analysis

### General Framework

**Figure 1:**
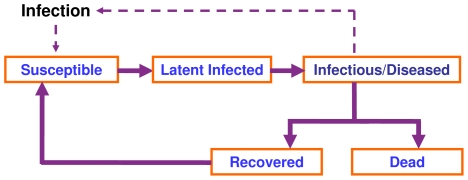
Model for transmission of bacterial or viral infections.

Consider a generic microparasitic disease in which individuals may move between infection states as illustrated in Figure 1. Upon exposure to infection a *susceptible* (i.e. not yet infected) individual may become infected and *infectious*, after which it may either recover or die. For simplicity, the states of *diseased* and *infectious* are considered equivalent in this study. The term *susceptible* does not indicate an individual's liability to infection; rather, it denotes that it is not immunologically resistant and can become infected. If *susceptible* individuals are replenished, either through loss of immunity of *recovered* individuals or through immigration of new individuals, then an endemic equilibrium may be reached in which the expected disease prevalence is constant. Otherwise, under assumptions of homogeneous random mixing the number of infected individuals will ultimately go to zero, and the epidemic will die out with the expected proportion of individuals ever infected during the course of the epidemic (I*) satisfying the equation 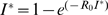 [12], where *R_0_* is the basic reproductive ratio of the disease. Therefore, assuming no disease-independent mortality, the expected proportion of susceptible individuals remaining in the population at the completion of the epidemic is 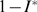.

Inferences about host genetic resistance are generally made by comparing *diseased* and *healthy* individuals. The *diseased* category will include infected and/or dead individuals, and the *healthy* category will include *susceptible* individuals, i.e. not yet infected, and possibly *recovered* individuals. In more complex models, individuals with latent infection that have yet to display detectable signs of infection may also be included in the *healthy* category. Heritabilities are determined by estimating to what degree the expected genetic relationships predict the classification of individuals into *healthy* and *diseased*, whereas individual SNP associations are inferred from departures of SNP allele frequencies from their expectations within the two categories. The genetic associations uncovered by such analyses will indicate host genetic variation in ‘disease resistance’, where the term ‘disease resistance’ is used generically to cover any of the processes shown Figure 1 that may influence the probability of an individual being diagnosed as *diseased*.

Several sources of uncertainty in field disease data can be identified from Figure 1. Firstly, for an individual to move from the *susceptible* to the *latently infected* or *infectious* category, it is necessary for it to be exposed to infection. A lack of exposure simply means that individuals do not have the opportunity to express their genotype for resistance, with potentially highly susceptible individuals being classified as *healthy*. In a group of individuals one might quantify exposure by *ε*, the probability that an individual is exposed to infection. Secondly, the diagnostic test used to classify individuals as *healthy* or *diseased* may be imperfect, with individuals misclassified. Specificity (*S_p_*) measures the probability that a healthy individual is classified as *healthy* by the diagnostic test, whereas sensitivity (*S_e_*) measures the probability that a diseased individual is classified as *diseased* by the test [11]. Thirdly, it is apparent from Figure 1 that an epidemic is a dynamic process. When data are collected over any time period which is less than the duration of the epidemic, the outcomes may differ from the outcomes that would have been obtained if the data were to have been collected over the entire epidemic, again through misclassifications.

These three phenomena whilst distinct are not independent, i.e. they are interrelated outcomes of the properties of the epidemic. For example, exposure probabilities may depend on the duration of data recording, with the probability of exposure increasing with time. However, for development of quantitative theory, their impacts are described and interpreted separately. The impacts of incomplete exposure and diagnostic test sensitivity and specificity can be explored independent of the epidemic dynamics, and hence are termed static disease properties. The impacts of time-dependent measurements require dynamic disease epidemic models.

### Static Disease Properties

#### (a) Incomplete Exposure to Infection

When there is incomplete exposure to infection the observed prevalence, the fraction of the whole population that is identified as *diseased* is a function of two factors: (i) the proportion of individuals that have been exposed to the pathogen (*ε*), and (ii) the virtual prevalence (*p*), which is defined as the proportion of individuals that have been exposed to the pathogen that become infected. Assuming that exposure is random and independent of host genotype, then the observed prevalence is 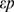. Of the 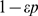 proportion of individuals that are *healthy*, 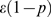 are exposed and apparently resistant, whilst 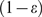 have not yet been exposed and have not expressed any genotype related to ‘disease resistance’. The phenotypic variance of observed ‘disease resistance’ is given by the binomial variance 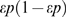.

Firstly, consider the epidemic among the exposed, with virtual prevalence *p*. Suppose that on the underlying liability scale the heritability is *h^2^* for true disease resistance, i.e. resistance following actual exposure, and the total liability has variance 1. Then using the linear approximation often used in the genetic analyses of binary traits [13], the genetic variance expressed on the binomial 0/1 scale is given by 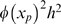 where *x_p_* is the truncation point of the Normal distribution corresponding to upper-tail probability *p*, and 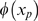 is the corresponding Normal density. Now consider the case of incomplete exposure and let *D′_u_* and *D′_w_* be the observed states (either *healthy*, 0, or *diseased*, 1) of individuals *u* and *w* with numerator relationship *a_uw_*, and let *Z* be an indicator trait with 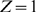 if both *u* and *w* are exposed and 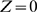, otherwise. Assuming exposure is independent of the numerator relationship then 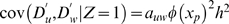 and 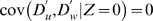, so 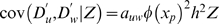; when *Z* = 0 the covariance is not expressed since at least one individual is not exposed, and there is only one outcome for that individual, *D*′ = 0. Then using the general formula for unconditional covariances: 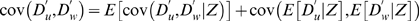 and noting (i) the latter term is 0, and (ii) 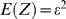 the probability of both being exposed, the result emerges: 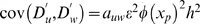.

Therefore on the 0/1 scale the true heritability of disease resistance is 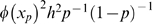 whilst the observed heritability is 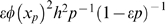. This differs by a factor 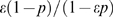. This will always be ≤1 since both *ε*≤1 and 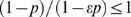. Furthermore, this biased heritability is transformed back to the liability scale as 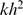, where 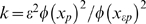. The bias on the liability scale is less than that on the observed scale since the reduced prevalence that is observed due to incomplete exposure leads to a greater scaling of the observed heritability back to the liability scale. For small 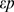, the under-prediction on the 0/1 scale is close to a linear function of *ε*. The bias is greater if *p* is moderate or large.

**Figure 2:**
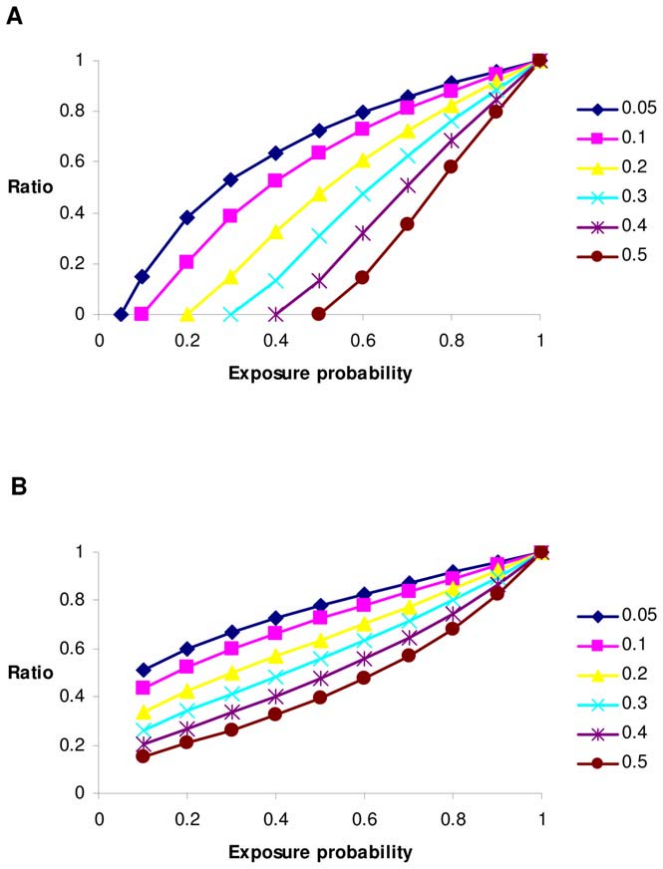
Ratio of estimated to true heritability on the liability scale for incomplete exposure. Results are shown for (A) differing observed prevalences or (B) differing virtual prevalences.

Impacts of differing exposure probabilities and differing virtual prevalences are illustrated in Figures 2a and 2b where observed and virtual prevalences are varied, respectively. In both cases the exposure probability has a close to linear impact on the bias parameter. The bias is more severe when considering the relationship as a function of observed prevalence, because when the exposure probability drops towards the observed prevalence, it implies the *healthy* population is dominated by individuals that have not been exposed to infection.

#### (b) Incomplete Sensitivity and Specificity of Diagnostic Tests

Individuals will be classified into *healthy* and *diseased* categories by means of a diagnostic test for the disease of interest. Fundamental to any diagnostic test are the concepts of specificity and sensitivity. As described above, specificity (*S_p_*) is the probability that a truly *healthy* individual is classified by the diagnostic test as *healthy* and sensitivity (*S_e_*) is the probability that a truly *diseased* individual is classified by the diagnostic test as *diseased*. The implications of sensitivity and specificity on the proportions of individuals diagnosed as healthy or diseased are shown in Table 1. The true prevalence is given as *p*, and the prevalence observed from the diagnostic test is *p*′.

**Table 1:** Proportions of individuals classified as Healthy or Diseased, as a function of Specificity (S_p_) or Sensitivity (S_e_).

		Classification by diagnostic test:		
		*Healthy*	*Diseased*	*Total*
True State:	*Healthy*	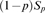	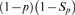	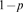
	*Diseased*	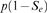	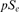	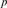
	*Total*	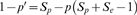	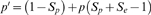	

Insight into the column margins can be gained by observing that 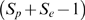 is the regression coefficient of the classification based upon the diagnostic test on the true state where disease is scored 1 and healthy 0. The regression line is 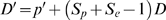. As above, let *D_u_* and *D_w_* be the true classification of individuals *u* and *w* with numerator relationship *a_uw_*. The impact of imperfect *S_e_* and *S_p_* on estimates of heritability can be deduced assuming that the classification errors are independent for *u* and *w*, and unrelated to *D_u_* or *D_w_*. The covariance between the observed classification *D′_u_* and *D′*_w_ can be obtained from 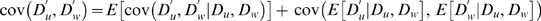. The first of these terms is identically zero given the assumption made. The second term is then the covariance of the terms in Table 2, which can be derived from the regression line above. This gives the result 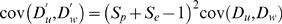. It then follows directly that if *u* and *w* have a genetic covariance of 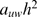 on the liability scale then 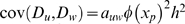 and 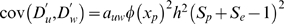 with observed prevalence *p*′. Thus, the observed heritability on the 0/1 scale is 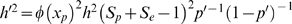 and when transformed back to the liability scale it is 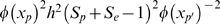.

**Table 2:** Covariance expectations between animals with different disease classification status.

*D_u_*	*D_w_*	Probability	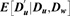	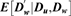
1	1	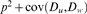	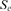	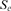
1	0	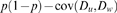	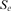	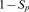
0	1	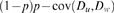	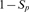	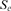
0	0	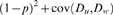	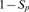	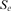

**Figure 3:**
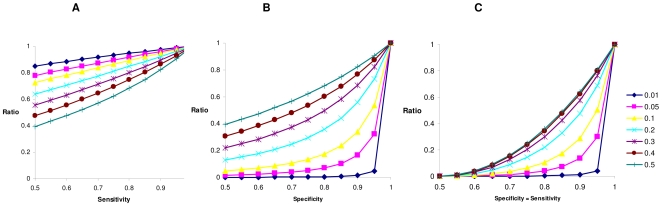
Ratio of estimated to true heritability on the liability scale for differing true prevalences. Results are shown for (A) incomplete sensitivity, where specificity = 1, (B) incomplete specificity, where sensitivity = 1 or (C) for incomplete specificity and sensitivity, where the two parameters equal.

Impacts of various specificities and sensitivities on estimated heritability values are illustrated in Figures 3a and 3b, where only sensitivity and specificity, respectively, are varied and 3c, in which they are varied jointly. For all prevalences, imperfect sensitivity and specificity both result in underestimated heritabilities on the liability scale. However the impact of poor specificities is much greater, for true prevalence less than 0.5. The reason for this difference is that when decreasing *S_e_*, the term 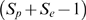 decreases, and the observed prevalence *p′* decreases also, so although 
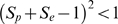
, this is partially compensated by 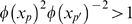. In contrast, when *S_p_* decreases, the observed prevalence *p*′ increases, and so both 
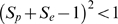
 and 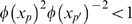. When both sensitivity and specificity are imperfect, then liability-scale heritabilities are considerably underestimated. This is likely to be the case in many practical situations, indicating that true genetic variation in disease resistance is likely to be much greater than indicated by analyses of field data.

### Dynamic Disease Properties

The principle of dynamic epidemic models is that individuals move between infection state categories, as shown in Figure 1. At different points during the epidemic it may be different individuals that are observably *diseased*, and the efficiency with which all potentially *diseased* individuals (I*) are observed as *diseased* depends on the duration of the data collection period in relation to the dynamics of the epidemic. In most data recording scenarios lasting for a time period *Δt*, i.e. temporally incomplete data recording, only a proportion of individuals ever transiting through the *infectious*/*diseased* categories will be observed. Let the total number of individuals observed to be *infectious*/*diseased* in the interval *t* to 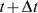 be defined as 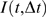 therefore the proportion of all individuals ever *diseased* that are observed is 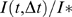. This is analogous to imperfect diagnostic test sensitivity. Therefore, the impact of temporally incomplete data recording on estimated heritabilities is the same as for imperfect sensitivity.

As an illustration of the impact of dynamic disease properties, consider a simple SIR model [12]. Let *S(u)* and *I(u)* be the instantaneous number of susceptible and infectious animals at time *u*, and *β* be the transmission coefficient for the disease. For a recording period starting at time *u = t*, and lasting for time period *Δt*, then 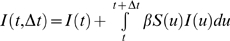. Therefore, the ratio 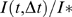 will depend not only on the duration of the recording period *Δt*, but also when recording commenced in relation to the epidemic. This ratio will be termed the ‘epidemic sensitivity’.

**Figure 4:**
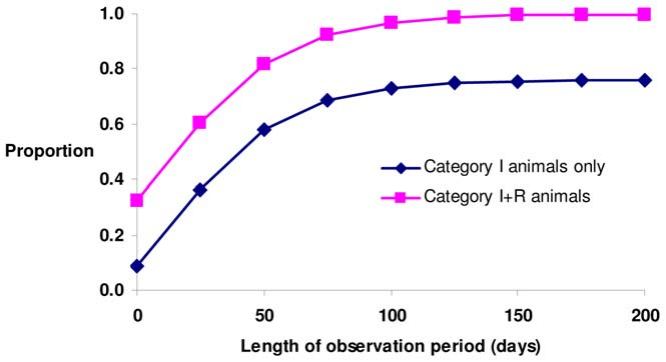
An example of the proportion of individuals recorded as *infectious/diseased* relative to those ever *infectious/diseased* during an SIR epidemic, as a function of recording period. Two cases are shown, with only I individuals observable or with both I and R observable. In this example, recording is triggered when prevalence reaches 5%. Parameters in this model are: *β* = 0.00015, *γ* = 0.1 and *R_0_* = 1.5.

As an illustration, consider an SIR model with parameters *β* = 0.00015, *γ* = 0.1, where *γ* is the recovery rate, *R_0_* = 1.5 and hence I* = 0.59. For this parameterization, and starting with one infected individual, it will take approximately 180 days for 95% of all individuals potentially infected during an epidemic to become *diseased*. It is assumed that recording starts when the disease prevalence reaches 5% and that the diagnostic test is perfect, i.e. sensitivity and specificity are both unity. Two scenarios are considered, (i) where only *infectious/diseased* individuals are observed, and (ii) where *recovered/removed*, e.g. dead, individuals are also observed. Plotted in Figure 4 are the proportions of individuals ever *diseased* during the course of the epidemic that are observed during the observation period, i.e. the epidemic sensitivity 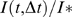. Observations taken only at one time point will result in a low epidemic sensitivity, hence underestimated heritabilities, and observations taken at different start points will also vary. If both *diseased* and *recovered/removed* individuals are observable, then the epidemic sensitivity becomes high with an extended observation period, since individuals that are infected and recover or removed prior to recording are also observed. However, if *recovered* individuals are not observable, i.e. they are healthy and no longer show any symptoms or clinical signs, then the epidemic sensitivity remains low and heritabilities remain underestimated.

## Discussion

This paper has provided a framework to assist in the interpretation of field disease data, with extensions to quantitative genetics theory being presented to account for the effects of various forms of environmental noise on genetic parameters for disease resistance. The factors considered, viz. incomplete recording, incomplete exposure, imperfect sensitivity and specificity of diagnosis are all typical of the non-genetic influences encountered with field disease data. We demonstrate in this paper that the likely impacts of these factors on genetic parameters for disease resistance are largely predictable, provided ball park figures can be obtained for specificity, sensitivity or exposure probabilities. In summary, estimable heritabilities are biased downwards by each of these factors. Conversely, the presence of detectable genetic variation in field disease data implies that the true heritability for disease resistance, were it to be measured under ideal circumstances, is likely to be much higher.

A further significance of the theory presented in this paper is that it can reconcile our observation that whilst traits describing immune responses to infection are often highly heritable, the disease outcomes that these traits influence tend to be lowly heritable. This is best illustrated from extensive datasets collected in farmed livestock. For example, components of innate and adaptive immunity are often moderately to highly heritable in commercial pig populations [14], [15], whereas the heritability of observable disease in such animals is low to moderate at best [16], [17]. Whilst true presence or absence of disease, given exposure to infection, will be largely a function of the immune response, we have demonstrated that the actual prevalence of disease and the estimable genetic variation between animals will be influenced by variable exposure and the sensitivity of diagnosis. Similarly, in commercial dairy cattle, many studies have demonstrated that the occurrence of clinical mastitis invariably has a heritability less than 0.1 [10], whereas underlying immune responses to infection display heritabilities which though variable are often high [e.g. 18].

**Figure 5:**
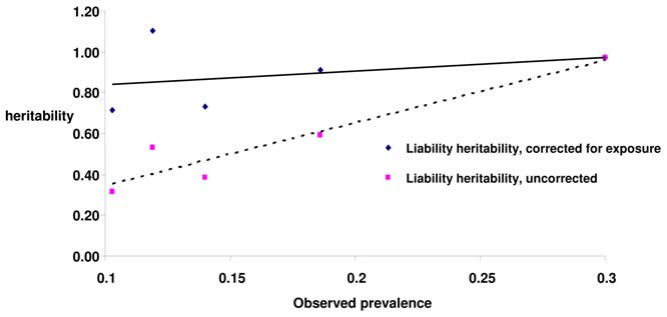
Heritabilities for liability to death from infectious pancreatic necrosis in five cohorts of Atlantic salmon, before and after correction for inferred relative exposure levels. The data are from Guy *et al.* 2009 [19]. Shown are heritability values and linear regression trend lines.

Published field data are available which supports the concepts developed in this paper. For example, predicted impacts of exposure to infection on estimable heritabilities may be inferred from data recently published on resistance to infectious pancreatic necrosis (IPN), a viral disease affecting farmed salmon. Heritabilities for IPN-related survival of salmon located in seawater localities containing the IPN virus were estimated and presented for seven independent cohorts of fish [19]. Of these seven cohorts, five fulfilled criteria of comprising populations unselected for IPN resistance and having heritability values consistent with the observed prevalence, i.e. heritabilities transformed to the liability scale [13] remained within the parameter space. For these five cohorts, the observed prevalences were 0.10, 0.12, 0.14, 0.19 and 0.30 and the corresponding heritabilities on the observed (0,1) scale were 0.11, 0.20, 0.16, 0.28 and 0.56, respectively, showing the expected strong relationship between prevalence and heritability for this scale. In principle, transformation to the liability scale should remove the relationship between prevalence and heritability, but the values obtained (0.32, 0.53, 0.39, 0.59 and 0.97) continue to show a significant linear relationship with prevalence. Because these five cohorts may be regarded as subpopulations sampled at random in relation to IPN resistance from the same overall population, it may be hypothesized that the differences in prevalence simply reflect differences in exposure rates. Relative exposure probabilities in each cohort may therefore be estimated as the ratio of observed prevalence to that seen in the cohort with the highest prevalence. Estimating exposure probability in this way, and using the above theory to rescale the heritability for liability, resulted in the heritabilities displayed in Figure 5, along with the regression of these heritabilities on observed prevalence. The strong linear relationship between prevalence and the heritability of liability to IPN disappeared when differences in relative exposure probabilities were hypothesized and the induced biases were removed. Furthermore it suggests that the heritability is large and important.

The heritability of resistance to bovine tuberculosis in dairy cattle provides an example of the potential impact of diagnostic test sensitivity and specificity on observable genetic variation. A recent publication provided convincing evidence of moderate genetic variation in tuberculosis resistance in dairy cattle, with an average heritability of liability of 0.12 in a dataset with a prevalence of 0.10 [9]; further, this paper speculated that imperfect sensitivity and specificity may have resulted in an underestimation of the true heritability. At this prevalence, imperfect specificity has a large impact on the estimated heritability, however the specificity of this diagnostic test is likely to be high. Sensitivity may be lower, possibly closer to 0.8 [20]. Exploring scenarios for specificities of 0.98 or 0.99, and sensitivities varying between 0.7 and 0.9, leads to the conclusion that the observed heritability is possibly underestimated by 20 to 40%. Therefore, the true heritability in this population is likely to be in the range 0.15 to 0.20.

Sometimes, particularly in an animal breeding context, an indicator trait is used to describe the impact of infection or disease upon an individual, for example somatic cell count in the milk of lactating ruminants with mastitis [10]. Hence, the measurements comprise a mixture distribution, i.e. those taken on both *healthy* and *diseased* individuals. These data may be analysed ignoring the fact that some individuals are *healthy* and others *diseased*, however this potentially leads to misleading results if the statistical properties of the trait (variance, heritability, etc) differ between the two subpopulations, or if the biological interpretation of the indicator trait differs between the two subpopulations. For example, dairy cattle breeders may wish to select on somatic cell count to reduce the incidence of mastitis, but they may not wish to alter mean somatic cell count in *healthy* cows [10]. Ideally, the data could be split into *healthy* and *diseased* subpopulations, and analysed separately. Various methods based on the properties of the data distribution have been proposed to achieve this [21]; alternatively an independent diagnostic of infection may be used, such as the presence of mastitis-causing microorganisms in the milk. Whatever approach is used, the concepts of diagnostic test accuracy still apply and biases may occur if these are ignored. For example the true difference in the indicator trait between the subpopulations will be underestimated for imperfect sensitivity or specificity, as animals will be misclassified.

We now determine the impact of imperfect sensitivity and specificity on the properties of indicator traits such as somatic cell count. If *H_i_* and *D_i_* are indicator trait observations in truly *healthy* or *diseased* subpopulations, and *H′_i_* and *D′_i_* are indicator trait observations in an imperfectly classified population in which the observed prevalence is *p*′, then the estimated true difference between diseased and healthy individuals 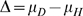 is, after simplification, 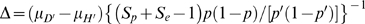. For plausible *S_p_* and *S_e_* values, *Δ* is always greater than 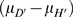. Similarly, properties of the variances of the observed subpopulations can be estimated from the properties of mixture distributions, and they contain an upwards bias proportional to *Δ^2^*. We have applied these concepts to mastitis in sheep (Riggio, Bishop and coworkers, unpublished data), using a dataset where diagnoses were available for the mastitis infection status of every ewe on every occasion that somatic cell count measurements were taken. These data demonstrated that specificity and sensitivity of diagnosis must have been high, as poor values would have led to implausible *Δ* values. Given high but plausible specificity and sensitivity (>0.9), inferred genetic correlations between the indicator trait measured in *healthy* and *diseased* animals were moderate (ca. 0.6) and insensitive to small changes in either parameter.

The theory presented in this paper does contain a number of simplifying assumptions, most notably that exposure probability or diagnostic test sensitivity and specificity are independent of host genotype. These assumptions may sometimes be violated. As an example, related individuals may be more likely to be co-exposed to infection, e.g. family members in the same household or animals in the same litter, and this potentially introduces a bias into heritability estimation. An issue may also arise with diagnostic tests in which animal immune responses are measured, such as skin test measurements used to infer exposure to bovine tuberculosis [20]. If aspects of these immune responses are genetic in origin, as seems plausible, this may impact on diagnostic test sensitivity. We have yet to fully explore the impact of these factors on expected genetic parameter values.

Many disease genetic studies now bypass the step of estimating variance components to quantify genetic variation and move directly to SNP association studies, unfortunately ignoring the design information that may give an objective assessment of the plausibility of both the design and the outcomes of the study. Nevertheless, the principles and consequences of noisy field data for the estimation of SNP effects are analogous to those for variance component estimation. For example, with incomplete exposure a fraction 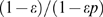 of individuals that are *healthy* have not been exposed and hence do not contribute information. Therefore, the effective size of the control population is smaller by this proportion. Furthermore, with imperfect sensitivity and specificity, there is a reduction in the estimable SNP effect size by 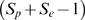 due to the regression coefficient of the diagnostic classification on the true state, with a consequent reduction in the experimental power for detecting SNP associations.

In summary, we believe that the results presented in this paper add clarity to the interpretation of field disease data, and reduce the risk that incorrect inferences are made regarding the extent of genetic variation. We have considered the different aspects of field data separately, but the underlying theory is clear and the potential exists to combine the different factors to match specific scenarios. We suggest that published estimates of heritabilities for resistance to microparasitic diseases, corresponding SNP effects and study design should be re-appraised given knowledge of the disease biology, i.e. likely exposure to infection, properties of the diagnostic tests and duration of data recording.
